# Efficiency of Field Laboratories for Ebola Virus Disease Outbreak during Chronic Insecurity, Eastern Democratic Republic of the Congo, 2018–2020

**DOI:** 10.3201/eid2901.221025

**Published:** 2023-01

**Authors:** Daniel Mukadi-Bamuleka, Fabrice Mambu-Mbika, Anja De Weggheleire, François Edidi-Atani, Junior Bulabula-Penge, Meris Matondo Kua Mfumu, Anaïs Legand, Antoine Nkuba-Ndaye, Yannick Tutu Tshia N’kasar, Placide Mbala-Kingebeni, John D. Klena, Joel M. Montgomery, Jean-Jacques Muyembe-Tamfum, Pierre Formenty, Johan van Griensven, Kevin K. Ariën, Steve Ahuka-Mundeke

**Affiliations:** Rodolphe Merieux INRB-Goma Laboratory, Goma, Democratic Republic of the Congo (D. Mukadi-Bamuleka);; Institut National de Recherche Biomédicale, Kinshasa, Democratic Republic of the Congo (D. Mukadi-Bamuleka, F. Mambu-Mbika, F. Edidi-Atani, J. Bulabula-Penge, M. Matondo Kua Mfumu, A. Nkuba-Ndaye, P. Mbala-Kingebeni, J.-J. Muyembe-Tamfum, S. Ahuka-Mundeke);; University of Kinshasa, Kinshasha (D. Mukadi-Bamuleka, F. Mambu-Mbika, F. Edidi-Atani, J. Bulabula-Penge, M. Matondo Kua Mfumu, A. Nkuba-Ndaye, P. Mbala-Kingebeni, J.-J. Muyembe-Tamfum, S. Ahuka-Mundeke);; Institute of Tropical Medicine, Antwerp, Belgium (A. De Weggheleire, J. van Griensven, K.K. Ariën);; World Health Organization, Geneva, Switzerland (A. Legand, P. Formenty);; Direction de la Surveillance Epidémiologique, Kinshasa (Y. Tutu Tshia N’kasar);; Centers for Disease Control and Prevention, Atlanta, Georgia, USA (J.D. Klena, J.M. Montgomery);; University of Antwerp, Antwerp, Belgium (K.K. Ariën)

**Keywords:** Ebola virus disease, EVD, Ebola, laboratory, Democratic Republic of the Congo, DRC, Institut National de Recherche Biomédicale, INRB, outbreak, chronic insecurity, viruses

## Abstract

During the 10th outbreak of Ebola virus disease in the Democratic Republic of the Congo, the Institut National de Recherche Biomédicale strategically positioned 13 decentralized field laboratories with dedicated equipment to quickly detect cases as the outbreak evolved. The laboratories were operated by national staff, who quickly handed over competencies and skills to local persons to successfully manage future outbreaks. Laboratories analyzed ≈230,000 Ebola diagnostic samples under stringent biosafety measures, documentation, and database management. Field laboratories diversified their activities (diagnosis, chemistry and hematology, survivor follow-up, and genomic sequencing) and shipped 127,993 samples from the field to a biorepository in Kinshasa under good conditions. Deploying decentralized and well-equipped laboratories run by local personnel in at-risk countries for Ebola virus disease outbreaks is an efficient response; all activities are quickly conducted in the field.

Since the discovery of Ebola virus in 1976, the Democratic Republic of the Congo (DRC) has faced 15 Ebola virus disease (EVD) outbreaks. The 10th outbreak was the longest (August 1, 2018, through June 25, 2020) and the most widespread and caused the most fatalities recorded to date in DRC (*1*,*2*). That outbreak occurred in urban–rural areas with high population mobility, was aided by good road infrastructure, and was driven by economic reasons and humanitarian issues. The movement of the population probably contributed to virus spread and created challenges for controlling the outbreak, in particular for contact tracing and ring vaccination. Community distrust of response teams, resulting from ≈2 decades of armed conflict in eastern DRC and commonly encouraged by sociopolitical leaders’ messaging, especially during the 2018–2019 election period (*3*), led to recurring misunderstandings and rejection of most countermeasures proposed to control the outbreak.

In response to the scale and mobility of this outbreak, the Institut National de Recherche Biomédicale (INRB) in Kinshasha, DRC, deployed 13 field laboratories and strategically positioned them across the outbreak areas (*3*). The laboratories were aimed at bringing diagnostic capacity closer to the outbreak epicenters, shortening turnaround times, and providing real-time guidance for newly available medical countermeasures. Most laboratories were provided with equipment and reagents to improve supportive care (*3*–*5*). Implementation and management required specific efforts, stringent biosafety measures, updated and well-maintained procedures, and technical expertise tailored to function in a volatile environment (*3*). We describe the deployment and management of field laboratories in terms of setup, logistics, technicalities, human resources, and security during the 10th EVD outbreak in eastern DRC.

## Deployment and Decommissioning of Field Laboratories

A field laboratory is a removable diagnostic unit, set up for specific purposes and for a limited time of operation (*6*,*7*). Field laboratories can be located in buildings adapted to the purpose or in transient structures or mobile platforms. In past EVD outbreaks, field laboratories were often established and managed through bilateral agreements between the host country and international entities. In DRC, a strong national EVD response and the expertise and availability of reverse transcription quantitative PCR diagnostic tools, GeneXpert Ebola assay (Cepheid, https://www.cepheid.com), ensured that the DRC INRB could coordinate and entirely manage laboratory response activities. Preparatory stages included strategic internal INRB meetings to discuss which type of laboratory to deploy.

During the 10th EVD outbreak, INRB deployed 3 types of laboratories: basic, standard, and advanced. The basic setup was focused on Ebola virus (EBOV) diagnosis, with a maximum of 2 GeneXpert instruments; biochemistry capacity was added if required. The standard setup had >2 GeneXpert instruments, along with biochemistry and hematology capacity for patient care and survivor follow-up (viral load in the body fluids, chemistry, and hematology). In the advanced setup, either differential diagnosis (Sudan or Bundibugyo ebolavirus, in addition to Marburg, dengue, chikungunya, yellow fever, West Nile, Crimean-Congo hemorrhagic fever, or Rift Valley fever viruses) or genomic sequencing capacity was added to the standard setup. Laboratory tests related to research activities, such as administration of EBOV therapeutics (*8*,*9*), immunogenicity of Ervebo vaccine (Ebola Zaire vaccine, https://www.fda.gov/vaccines-blood-biologics/ervebo), and studies of EBOV reservoirs in bats were conducted in standard and advanced setups. After internal discussions, a proposal was presented to the response coordination team to collect feedback, if any. If agreement was reached, the laboratory was deployed according to INRB checklists for equipment, reagents, and accessories (Appendix). Materials were transported by air, land, or water with >1 INRB staff member assisted by local laboratory personnel. On site, the team set up the laboratory by adapting the workflow to the existing rooms or by rehabilitating or building additional space, if needed. In general, field laboratories were housed in structures located as close as possible to the Ebola treatment centers that they serviced. The average time between decision making and laboratory deployment was 4.7 (range 1–10) days. After deployment, the laboratory was functional within 24 hours. Thirteen field laboratories were deployed in the 3 affected provinces: 6 basic, 4 standard, and 3 advanced laboratories (Table 1). The laboratories of Beni, Mangina, and Goma were deployed from Kinshasa; the others were deployed from Beni (the main field laboratory); 1 laboratory (Tchowe, South-Kivu) was deployed from Goma field laboratory (Table 2). 

**Table 1:** Type of setup, activities, and sites in field laboratories for EVD outbreak, eastern Democratic Republic of the Congo, 2018–2020*

Type of laboratory setup	Activities performed	Sites/provinces
Basic	EVD diagnosis: GeneXpert (+)†; Chemistry: Piccolo (+/–)‡	Tchomia/Ituri, Bunia/Ituri, Tchowe/South-Kivu Bukavu/South-Kivu, Biakato/Ituri, Kasindi/North-Kivu
Standard§	EVD diagnosis: GeneXpert (+); Chemistry: Piccolo (+); Hematology: pocH-100i (+)¶; Ebola survivors clinic	Mangina/North-Kivu, Goma/North-Kivu, Komanda/Ituri, Mambasa/Ituri
Advanced§	EVD diagnosis: GeneXpert (+); Chemistry: Piccolo (+); Hematology: pocH-100i; Ebola survivors clinic; EVD differential diagnosis (Smart cycler or RPA) (+) or genomic sequencing (+)	Beni/North-Kivu, Butembo/North-Kivu, Katwa/North-Kivu

*EVD, Ebola virus disease; RPA, recombinase polymerase amplification. †GeneXpert, (Cepheid, https://www.cepheid.com). ‡Abaxis (https://www.abaxis.com). §Sysmex (https://www.sysmex.com). ¶Research support: Ebola therapeutics through a Monitored Emergency Use of Unregistered and Investigational Interventions (MEURI) protocol or the Pamoja Tulinde Maisha/Together save lives (PALM) randomized controlled trial; the immunogenicity of rVSV-ZEBOV-GP vaccine; and the study of Ebola virus reservoir in bats.

**Table 2:** Origin of field laboratories deployed over time, distance covered, and transport means used, eastern Democratic Republic of the Congo, 2018–2020

Origin of deployment	Laboratory	Distance traveled, km	Means of transport
Kinshasa	Beni	1,669	Airplane
Kinshasa	Mangina	1,692	Airplane/car
Kinshasa	Goma	1,580	Airplane
Beni	Butembo	54	Car
Beni	Tchomia	256	Helicopter
Beni	Bunia	147	Helicopter
Beni	Katwa	60	Car
Beni	Komanda	127	Helicopter
Goma	Tchowe	421	Helicopter
Beni	Bukavu	339	Airplane
Beni	Mambasa	137	Helicopter
Beni	Biakato	70	Helicopter
Beni	Kasindi	75	Car

Throughout the outbreak, there were multiple epicenters, which changed in intensity and locations over time. Deploying a flexible model of laboratories at the epicenters of the outbreak enabled well-structured and coordinated actions involving all pillars of the response. Results were quickly provided on site to enable timely public health interventions and mitigate the risk for security incidents as transportation of samples was reduced. Decentralized setups were maintained to support routine EVD surveillance in remote areas and detect eventual flare-ups. The presence of skillful local laboratory workers strongly decreased the number of national and foreign experts to be deployed and fostered community engagement. Field laboratories helped integrate response activities into the health system.

Seven laboratories were then decommissioned. All instruments were decontaminated according to INRB standard operating procedures, disassembled, and packed in specific boxes or suitcases, shipped to Beni, and later shipped to Goma or Kinshasa. The laboratories in Beni, Mangina, Butembo, Bunia, Bukavu, and Mambasa are still functional.

## Field Laboratory Composition, Biosafety and Biosecurity, and Cleaning and Decontamination 

**Figure 1:**
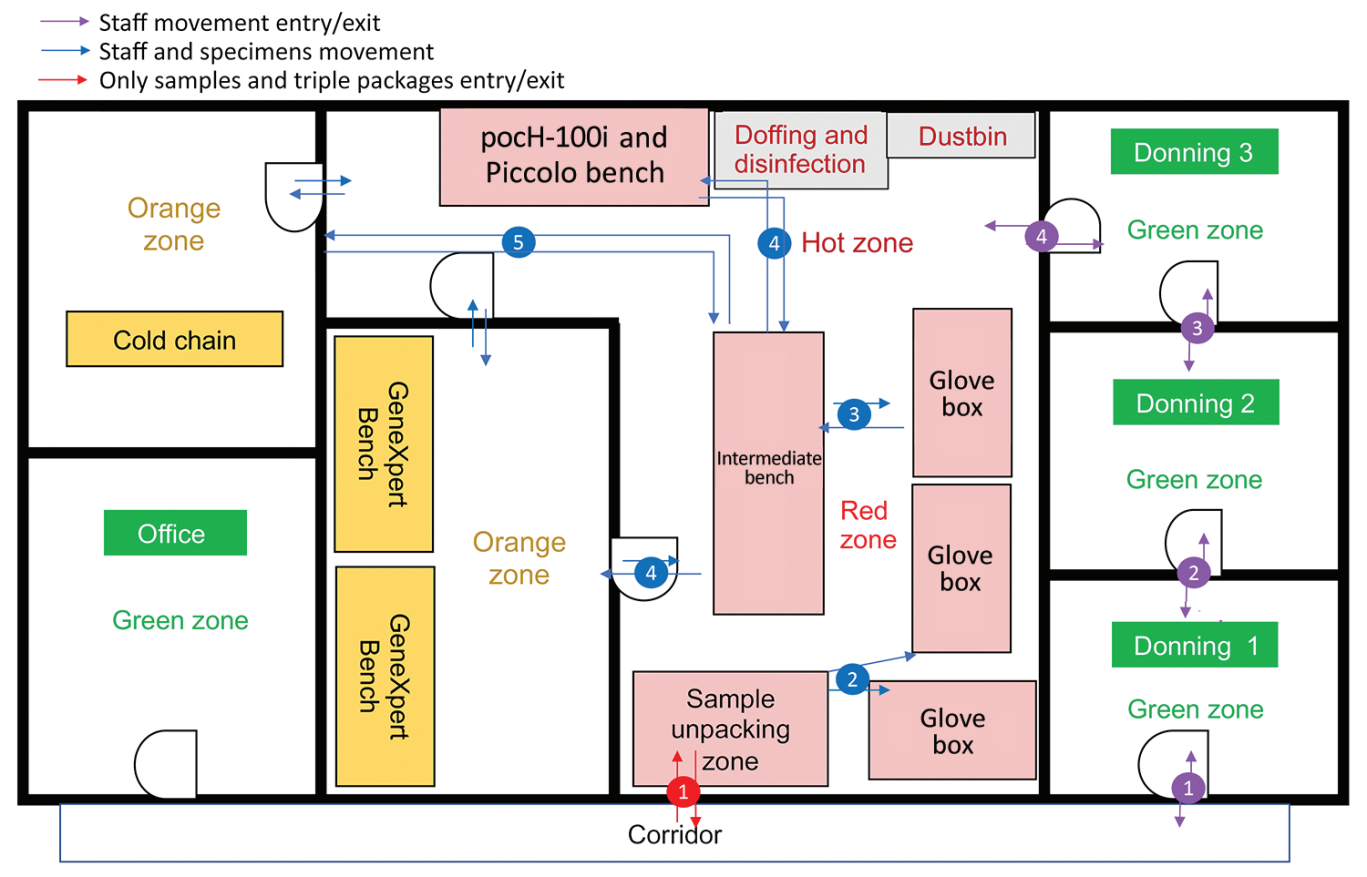
Advanced field laboratory setup used for Ebola virus disease outbreak during chronic insecurity, eastern Democratic Republic of the Congo, 2018–2020. GeneXpert, (Cepheid, https://www.cepheid.com); pocH-100i (Sysmex (https://www.sysmex.com); Piccolo (Abaxis (https://www.abaxis.com).

**Figure 2:**
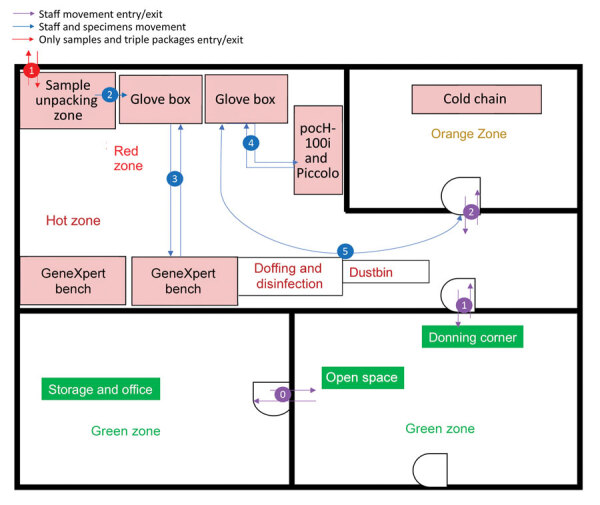
Basic field laboratory setup used for Ebola virus disease outbreak during chronic insecurity, eastern Democratic Republic of the Congo, 2018–2020. GeneXpert, Cepheid, https://www.cepheid.com; pocH-100i, Sysmex, https://www.sysmex.com; Piccolo, Abaxis, https://www.abaxis.com.

To mitigate the risk for exposure, protect the safety of the laboratory workers, and prevent environmental contamination, EBOV-suspected samples must be handled in a Biosafety Level 3 (BSL-3) laboratory or BSL-3–like conditions (standard personal protective equipment [PPE], negative pressurized glove box, restricted access to the laboratory, laboratory staff trained and vaccinated) (*10*,*11*). Hence, deployment of BSL-3–like structures is needed at outbreak locations. An INRB field laboratory contained 3 mandatory areas: hot zone, cold zone, and extra space. Inside the hot zone, we considered the red zone as the space for sample reception, unpacking and testing (Piccolo [Abaxis, https://www.abaxis.com], pocH-100i [Sysmex, https://www.sysmex.com], and iStat [Abbott, https://www.abbott.com]), and PPE doffing; the orange areas were set up for GeneXpert bench (Cepheid, https://www.cepheid.com) and cold chain. The cold zone included multiple-step donning areas. The extra space was used for supply storage and the administrative office (Figure 1). The basic setup had a hot zone and an extra space in which donning PPE, supplies storage, and administrative offices were located (Figure 2). Samples for diagnosis were inactivated and aliquoted within the glove box. Piccolo discs and iStat cartridges were prepared in the glove box and tested with respective instruments at the bench. Because the red and orange zones were contiguous in most settings, the staff working in these areas dressed in full PPE (Tyvek coverall, goggles or face shield, mask [N100 or FFP3], long-cuff nitrile gloves, and laboratory shoes with shoe covers). In cold zones, staff were dressed with light PPE (surgical gown, head covering, and mask [N95 or FFP2]; short-cuff nitrile gloves; and laboratory shoes). Access to the laboratory was restricted to INRB-trained and Ervebo-vaccinated persons (vaccinated >10 days before working).

At the beginning of the day, trained hygienists cleaned the floors with 0.5% bleach and emptied the dustbins while dressed in full PPE (hot zone) or light PPE (cold zone). At the end of the day, laboratory operators disinfected the inner part of the glove boxes and the benches with bleach 0.5% or other solutions (e.g., Rely+On [Virkon, https://relyondisinfection.com], CDiffend [2XL Corporation, https://www.2xlpro.com]), according to INRB standard procedures. In addition to daily cleaning/decontamination, every 2 weeks in each laboratory, workers decontaminated the glove boxes (inside/outside), benches, and all other surfaces and, maintained instruments. Cleaning/decontamination activities were recorded in laboratory registers, maintenance sheets, and decontamination sheets.

## Field Laboratory Equipment

### Point-of-Care Devices

Laboratory capacities evolved over the course of multiple EVD outbreaks and followed the level of care provided to persons with confirmed cases. In the past, most EVD laboratories had only reverse transcription PCR testing. Toward the end of the 2013–2016 outbreak in West Africa, cost-effective, rapid, and sensitive tools to detect EBOV at point-of-care and to guide provision of treatments had been developed but were not widely available (*12*). During the ninth EVD outbreak in DRC (Equateur, 2018), the GeneXpert technology was introduced in a structured way to diagnose EVD in 3 remote laboratories. The tool enables a quick diagnosis (turnaround time <4 hours) and differentiates, by means of its double target (nucleoprotein [NP] and glycoprotein [GP]), between recently Ervebo-vaccinated persons (only GP gene detected) and persons with acute EVD cases (NP and GP detected). From the 10th EVD outbreak on, a standard Ebola field laboratory included in addition to GeneXpert, pocH-100i (hematology), Piccolo (biochemistry), i-STAT (biochemistry), and a glove box (virus inactivation and sample processing).

A total of 47 GeneXpert IV-modules, 17 Piccolo, 10 iStat, 8 pocH-100i, and 19 glove boxes were deployed. Three types of glove boxes were used: 8 Cleatech HEPA Filtered (Cleatech LLC, https://www.cleatech.com), 8 Könnecke Ultra (Bodo Könnecke, https://www.koennecke-berlin.de), and 3 Germ Free (Germfree Laboratories https://www.germfree.com) (Table 3).

**Table 3:** Distribution of equipment per field laboratory for Ebola virus disease outbreak, eastern Democratic Republic of the Congo, 2018–2020*

Equipment	Beni	Goma	Butembo	Mangina	Katwa	Kasindi	Tchomia	Bunia	Komanda	Biakato	Mambasa	Bukavu	Tchowe	Total
GeneXpert†	10	4	9	5	4	2	1	2	3	2	3	1	1	47
Piccolo‡	3	1	3	3	2	0	1	0	1	1	1	0	1	17
iSTAT§	3	1	2	2	0	0	0	0	0	0	1	0	1	10
pocH-100i¶	2	1	1	1	1	0	0	0	1	0	1	0	0	8
Glove box	4	2	2	1	2	1	1	1	1	1	1	1	1	19
Refrigerator, +2°C–+8°C	5	1	1	2	1	2	1	1	1	1	1	1	1	19
Freezer, –20°C	5	1	1	0	1	1	1	1	1	1	1	0	0	14
Freezer, –40 and –80°C	9	0	0	0	0	0	0	0	1	0	0	0	0	10
Laptop	6	1	2	3	2	1	0	1	1	1	1	2	0	21
Printer	5	1	2	1	2	1	0	1	2	1	1	1	1	19
Smart cycler	1	0	0	0	0	0	0	0	0	0	0	0	0	1
RPA	1	0	1	0	0	0	0	0	0	0	0	0	0	2
Total	54	13	24	18	15	8	5	7	12	8	11	6	6	186

*RPA, recombinase polymerase amplification. † Cepheid, https://www.cepheid.com. ‡Abaxis, https://www.abaxis.com. §Abbott, https://www.abbott.com. ¶Sysmex, https://www.sysmex.com.

### Maintenance and Quality Control

Instrument maintenance was performed every 2 weeks, according to INRB standard procedures. Quality control was done monthly on instruments per site by using the Piccolo control kit level 1, 2, 3; the iStat TriControls level 1, 2, 3; the Sysmex Eightcheck3WP-N/L/H; and a GeneXpert positive blood and semen specimen (prepared at the US National Institutes of Health, National Institute of Allergy and Infectious Diseases, Bethesda, Maryland, USA). Xpert calibration cartridges were run on all instruments every 6 months. All records were stored in specific binders in the laboratory and uploaded on INRB dropbox and Huddle cloud (https://us.huddle.com; no longer available).

### Cold Chain Setup

The standard cold chain included refrigerators (+2°C to +8°C) and freezers (–20°C). The main cold chain located in the Beni laboratory could store up to 40,000 specimens at a time (from different laboratories). It comprised refrigerators and 3 types of freezer (–20°C, –40°C, and –80°C) (Table 3). Power was supplied by three 14-kva generators running alternately, 24 hours/7 days a week. In other laboratories, standard cold chain was continuously connected to five 10–kva generators. Site-to-site transport of specimens was achieved by using biological triple-packaging boxes (UN 4H2/GLASS 6.2/14 or BioPack-2 [Airs Sea Containers LTD, https://www.airseadg.com]) filled with frozen icepacks. Given the limited storage capacity in the main cold chain, samples were shipped to the biorepository in Kinshasa. In each laboratory, samples were stored at –20°C and reagents at +2°C to +8°C. Traceable Otio (https://www.otio.com) trackers were used to monitor the temperature inside refrigerators, freezers, and laboratory spaces twice daily. Approximately 120,000 samples were stored in the field and then shipped safely to the Kinshasa biorepository for long-term preservation (*3*).

## Logistics for Implementing and Managing Field Laboratories

During the 10th EVD outbreak, field laboratories faced tremendous challenges. The 7 main challenges were: 1) activities interruption after attacks on response teams; 2) movement of contacts and suspected and confirmed case-patients resulting in further spread of the disease; 3) slow resumption of activities after security incidents; 4) evacuation of response staff out of outbreak areas during insecurity events; 5) delayed implementation of activities; 6) disruption to refilling laboratory supplies and fuel; and 7) delayed sample transportation to the laboratory (especially for sequencing unit) (*13*).

### Equipment and Supplies

Most equipment and supplies were purchased abroad and shipped to Kinshasa. Thereafter, they were airlifted to Beni laboratory, from where the distribution was organized to other sites. Parcels were transported by road, air, and boat by using the World Health Organization and the World Food Program logistics. Finale Inventory software (https://app.finaleinventory.com) was used for the stock management of items at the main warehouses in Beni and Kinshasa. It alerted the laboratory and logistics staff via email regarding the status of items (in critical stock or nearly to be expired). The logistics team could therefore anticipate shortages, prepare timely orders, and prioritize the use of items close to expiration date.

## Laboratory Activities

The most prominent activities conducted in the field laboratories were EVD diagnosis and training. Laboratories contributed to providing investigational therapeutics to treat patients with confirmed cases, and they were equipped with clinical laboratory capacities (*8*,*9*). Five laboratories supported survivor activities, 1 conducted genomic sequencing, and 2 performed differential diagnoses (*13*,*14*). Throughout the outbreak, 9.3 tons of equipment were deployed across sites (*3*), 230,936 Ebola Xpert cartridges were used and subsequently shipped to the Goma laboratory for proper disposal by incineration, as per World Health Organization recommendations. Laboratory performance was evaluated by 2 indicators: proportion of new suspect samples tested within 48 hours and proportion of results delivered within 24 hours. Despite challenging conditions, field laboratories tested 100% of samples with turnaround times of <48 hours.

### Sample Management, Sample Testing, and Results Communication

Samples were collected by the surveillance team; care team; safe and dignified burials team; or survivors’ team. The preferred sample for EBOV diagnosis was whole blood or plasma, although other specimens were oral secretions from cadavers, blood swab samples (patients with circulatory collapse, infants), semen and vaginal secretions from survivors, fetal annexes in delivering survivors, and breast milk in breastfeeding survivors. In care units, samples were collected 1–4 hours after admission during the day (delay mainly resulted from time to stabilize the patient, high number of patients to be sampled, and preparation of sampling material and notification forms). Patients received at night underwent sampling the next morning, after the laboratory was opened. Samples were transported in triple-packaging boxes to the laboratory within 10–20 minutes. At the laboratory, samples were received on a dedicated table, unpacked while personnel dressed in PPE disinfected different layers of the packing, and then transferred through a window into the hot zone for processing.

Chemistry and hematology samples were given priority because those results could help clinicians quickly adjust the care provided (e.g., correction of hypoglycemia, electrolyte imbalance, anemia, shock). Chemistry and hematology samples were processed within 30–40 minutes (from reception to testing). The Piccolo results were read within 12 minutes, whereas pocH-100i outputs were available in 2 minutes. For diagnosis, new suspected samples were processed first to confirm or invalidate their EVD status, and then the follow-up samples were processed. Only the amount of sample required for testing in the GeneXpert lysis buffer (guanidinium thiocyanate) was inactivated. Samples for hematology and chemistry were not inactivated. Cryotubes were labeled (initials of the site, number assigned in an ascending way, type of sample, and type of blood tube), samples were aliquoted in cryotubes, and then aliquots were transferred into 9 × 9 labeled grids (same type of samples per cryobox) and stored in the cold chain.

### Handling Data and Communicating Results

Samples tubes were identified per team collecting the specimen. A notification form accompanying the sample to the laboratory contained the patient identification and sociodemographic, epidemiologic, and clinical information. Additional information (laboratory identification, type of sample, analyses done, type of blood tube, number of aliquots, grid labels, date of testing, EBOV results, and cycle threshold values) were added in the laboratory to make a line list in Microsoft Excel 2016 (https://www.microsoft.com). All issues related to spelling or transcription errors were deconflicted by using sociodemographic, clinical, epidemiologic information, and laboratory results. The communication of laboratory results was organized by psychosocial teams in close coordination with families and other teams (*3*). The laboratory head shared results daily with the response coordination via sending the Microsoft Excel line list by email. All laboratory results were centralized in a database (*15*). Most samples (212,655 [89.1%]) were tested with GeneXpert, 19,227 (8%) by clinical chemistry, and 6,766 (2.8%) by hematology. In total, 207,065 (86.8%) blood, 27,313 (11.4%) oral swabs, and 1,544 (0.6%) semen samples were tested (Table 4). We were successful in testing this unprecedented number of samples because of the extended duration of the outbreak (22 months), the number of laboratories deployed, the diversity of activities performed (diagnostic, patient biochemistry and hematology, viral load monitoring, survivor follow-up, and full-genome sequencing), the management of all laboratories by 1 institution (centralized information, data, and samples), and the community-based surveillance (which improved sample flow).

**Table 4:** Types of analyses per field laboratory site, eastern Democratic Republic of the Congo, 2018–2020*

Laboratory	Analyses performed					Ebola RT-PCR confirmation			Sample type					
	RT-PCR	Bio	Hemat	Total		Positive result	Samples received		Blood	Oral swab	Vaginal secretions	Sperm	Other*	Total,
Beni	62,706 (82.3)	10,612 (14)	2,850 (3.7)	76,168 (31.9)		924 (1.5)	61,690		68,004 (89.2)	7,035 (9.2)	524 (0.6)	432 (0.6)	173 (0.2)	76,168
Butembo	69,027 (90)	5,349 (7)	2,357 (3)	76,733 (32.1)		1,129 (1.7)	67,142		64,788 (84.4)	9,721 (12.6)	1,189 (1.5)	723 (1)	312 (0.4)	76,733
Mangina	21,656 (93)	980 (4.2)	665 (2.8)	23,301 (9.7)		728 (3.5)	21,077		19,511 (83.7)	3,199 (13.7)	253 (1)	327 (1.4)	11 (0.04)	23,301
Katwa	34,244 (94.8)	1,140 (3.1)	715 (2)	36,099 (15.1)		542 (1.6)	34,244		32,266 (89.3)	3,825 (10.5)	2 (0)	2 (0)	4 (0)	36,099
Kasindi	5,032 (100)	0	0	5,032 (2.1)		0	5,032		4,556 (90.5)	466 (9.2)	0	0	10 (0.2)	5,032
Tchomia	125 (91.2)	12 (8.7)	0	137 (0.05)		2 (1.7)	115		125 (91.2)	10 (7.2)	0	0	2 (1.4)	137
Komanda	7,522 (92.5)	541 (6.6)	65 (0.7)	8,128 (3.4)		106 (1.4)	7,522		5,882 (72.3)	2,077 (25.5)	0	0	169 (2)	8,128
Biakato	1,733 (98.5)	26 (1.4)	0	1,759 (0.7)		20 (1.2)	1,733		1,446 (82.2)	313 (17.7)	0	0	0	1,759
Mambasa	10,176 (93.8)	552 (5.1)	114 (1)	10,842 (4.5)		36 (0.4)	9,687		10,062 (92.8)	643 (6)	76 (0.7)	60 (0.5)	1 (0)	10,842
Tchowe	434 (96.6)	15 (3.3)	0	449 (0.1)		5 (1.2)	434		425 (94.6)	24 (5.3)	0	0	0	449
Total	212,655 (89.1)	19,227 (8)	6,766 (2.8)	238,648 (100)		3,492 (1.7)	208,676 (100)		207,065 (86.8)	27,313 (11.4)	2,044 (0.9)	1,544 (0.6)	682 (0.3)	238,648

*Values are no. (%). Bio, biochemistry; hemat, hematology; RT-PCR, reverse transcription PCR.

### Sample Shipments

Most samples collected were shipped to the INRB biorepository in Kinshasa along with corresponding databases by email. Shipments of large batches of samples were done with chartered cargo flights. Site-to-site sample transportation was performed by fleets and vehicles supported by the World Food Program, MONUSCO (https://monusco.unmissions.org), and the World Health Organization. A total of 127,993 samples were shipped from the Beni laboratory to Kinshasa through 7 large shipments. Decision making for samples shipment was guided by the limitation of the storage capacity on the ground and the need to evacuate the Ebola specimens to a safe area.

## Human Resources Management 

**Figure 3:**
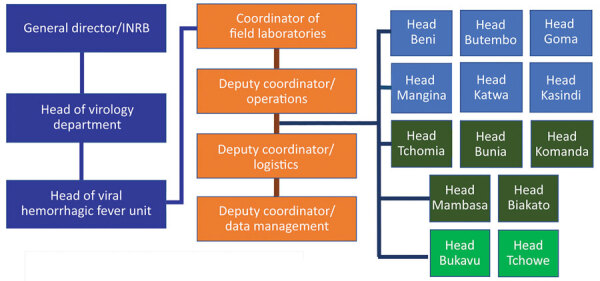
Organizational chart for field laboratories used for Ebola virus disease outbreak during chronic insecurity, eastern Democratic Republic of the Congo, 2018–2020. INRB, Institut National de Recherche Biomédicale.

Activities were conducted by medical-biologist doctors, biologists, and laboratory technicians assisted by supporting staff (administrative, logistics, hygienists, security guards, drivers) (Table 5; Figure 3). Local staff members were recruited mostly in the main health facilities of affected provinces or cities; national staff came from INRB (Kinshasa). All laboratory personnel were trained with regard to biosafety and biosecurity; PPE donning and doffing; good clinical and laboratory practices; sample collection, packaging, and transportation; sample manipulations within a glove box; instrument manipulation and troubleshooting; and safety and emergency management. Refresher trainings were scheduled each trimester, whenever a new staff member started at the laboratory or when an error occurred throughout the testing process. The laboratory was headed by a medical-biologist doctor or biologist assisted by a bench supervisor. The laboratory head was responsible for sample processing, results validation and delivery, quality control and final laboratory records, participation in daily coordination meeting, engaging with other working groups, and drafting the reports and communication. The laboratory head reported daily to the coordinator of all field laboratories who, in turn, presented the global situation at the general coordination meeting. Staff were granted a break after 3 months of work or in any emergency situation. The bench supervisor was responsible for organizing the bench workload and schedules, sample processing and storage, functioning and maintenance of laboratory instruments, cleaning and decontamination procedures, records tracking, and training of new staff. The administrative staff were in charge of preparing paperwork, data entry, data management, results typing, printing, and distribution. Over the course of the outbreak, INRB created a unique database template used by all laboratories, which were shared monthly with the coordinator of field laboratories. We employed 134 personnel, including 9 medical-biologist doctors, 21 biologists, and 62 laboratory technicians (Table 5). INRB fostered capacity building to quickly hand over Ebola response tools and competences to local staff to empower the health system and decentralize the diagnostics. The local capacity setup at the peripheral level allowed sustained and successful management of 3 successive EVD flare-ups (Butembo in 2021, Beni in 2021 and 2022).

**Table 5:** Field laboratory staff deployed or recruited, eastern Democratic Republic of the Congo, 2018–2020*

Staff type	Beni	Goma	Butembo	Mangina	Katwa	Kasindi	Tchomia	Bunia	Komanda	Biakato	Mambasa	Bukavu	Tchowe	Total no. (%)
Laboratory technician	7	6	12	5	1	6	1	3	4	3	4	7	3	62 (46,2)
Medical biologist	5	3	3	1	3	0	0	1	1	0	1	3	0	21 (15,6)
Data manager	2	1	2	1	1	1	0	1	1	1	1	1	0	13 (9,7)
Medical-biologist doctor	3	0	3	1	1	0	0	0	0	1	0	0	0	9 (6,7)
Hygienist	1	1	1	1	1	1	1	1	1	1	1	1	1	13 (9,7)
Phlebotomist	0	0	0	0	8	0	0	0	0	0	0	0	0	8 (5,9)
Logistician	4	0	1	0	0	0	0	0	0	0	0	0	0	5 (3,7)
Administrative	2	0	0	0	0	1	0	0	0	0	0	0	0	3 (2,2)
Total no. (%)	24 (17.9)	11 (8.2)	22 (16.4)	9 (6.7)	15 (11.1)	9 (6.7)	2 (1.4)	6 (4.4)	7 (5.2)	6 (4.4)	7 (5.2)	12 (8.9)	4 (2.9)	134

## Security Concerns during the 10th EVD Outbreak

The overall societal context of the eastern DRC led to express reluctance toward most EVD countermeasures. Of the 13 laboratories, 10 were deployed within unsafe areas. Thus, on several occasions, teams were attacked by rebels, militia, and other hostile groups. Those threats delayed or impeded deployment and supervision of activities, sample handling, and staff movement (Tables 6, 7). To manage security in the field, transportation of all personnel movement, equipment, and supplies had to be approved by a security commission. Depending on the context, some convoys had to be escorted by security forces, using military devices such as armored vehicles and pickup trucks with machine guns and bulletproof vests for passengers. In unsafe areas, a curfew was set from 6:00 pm to 6:00 am, except for a few teams, which were circulating under security escort until late to maximize timely results. In unsafe areas, laboratories were protected by armed guards to prevent eventual attacks, sabotage, or disruption. Despite the security incidents encountered, field laboratories operated for an average of 21 months, supporting all pillars of the response.

**Table 6:** Armed groups in Ebola operational zones and time taken to deploy and operate field laboratory, eastern Democratic Republic of the Congo, 2018–2020*

Site	Security background	Results achieved		
		Date of installation	Time taken to deploy laboratory	Time laboratory remained functional, mo
Beni	ADFrebels: near Virunga Parc (ADF stronghold); popular pressure groups and citizens movements; Maï Maï rebel groups	2018 Aug 2	1 d	45*
Mangina	Maï Maï rebel groups; ADF rebels	2018 Aug 11	9 d	45*
Butembo	Maï Maï rebel groups; popular pressure groups; citizens movements	2018 Sep 7	6 d	44*
Tchomia	Maï Maï rebel groups; CODECO rebel Group	2019 Sep 28	6 d	3
Katwa	Maï Maï rebel groups; popular pressure groups	2019 Jan 18	10 d	26
Komanda	Maï Maï rebel groups; ADF rebels	2019 Jan 18	7 d	15
Mambassa	Maï Maï rebel groups; ADF rebels	2019 Sep 2	10 d	18
Tchowe	Maï Maï rebel groups; FDLR rebels	2019 Aug 19	2 d	2
Biakato	Maï Maï rebel groups; ADF rebels	2019 Oct 12	10 d	5
Kasindi	Located in the Virunga Park (Ugandan border):; Ugandan rebels stronghold	2019 Oct 28	10 d	6

*As of May 2, 2022. ADF, Allied Democratic Forces; CODECO, Coopérative pour le Développement du Congo; FDLR, Democratic Forces for the Liberation of Rwanda.

**Table 7:** Major security incidents during the 10th Ebola virus disease outbreak, eastern Democratic Republic of the Congo, 2018–2020

Incident	Site (period)	Consequences
Attack on the response team	Beni (Aug 2018)	Response activities stopped, and recovery took longer to reach full operation
Several days off + murder of civilians	Beni (Sept 2018)	Response activities stopped for few days
Election unrest + destruction of the ETU	Beni/Butembo (Dec 2018)	Response teams evacuated
Attacks on laboratory, surveillance, and coordination teams	Oicha-Eringeti (Dec 2018)	Komanda laboratory deployment delayed
Attackers set fire to Katwa and Butembo ETU	Katwa/Butembo (Feb 2019)	Temporary ETU closure
Attack on the response teams	Butembo (Apr 2019)	Response activities stopped, and 1 foreign doctor killed
Attack on the response teams	Biakato/Beni (Nov 2019)	Evacuation of teams to Goma and Kinshasa, 6 deaths

*ETU, Ebola treatment unit.

## Conclusions

In countries at high risk for EVD outbreaks, decentralized laboratories should be strategically positioned to timely detect EBOV. Health authorities should supply them with dedicated equipment and well-prepared teams, built on local know-how to organize efficient responses. A tiered and decentralized policy of laboratories during outbreaks provides flexibility for managing materials and supplies, logistics, and human resources in the health system. The quick handover of competences and capacities to local staff led during the 10th EVD outbreak in the DRC led to sustained and successful management of further outbreaks. Nonetheless, efforts should be made to use new diagnostic and research tools in the laboratory to considerably reduce turnaround time to <24 hours, to strongly improve patient management and research activities, to foster real-time genomic sequencing, and to support follow-up of EVD survivors.

Appendix Additional information for deployment, management, and performance of field laboratories for Ebola virus disease outbreak during chronic insecurity, eastern Democratic Republic of the Congo, 2018–2020.
