# Supplementary material for: Efficiency of Field Laboratories for Ebola Virus Disease Outbreak during Chronic Insecurity, Eastern Democratic Republic of the Congo, 2018–2020
Source: Emerg Infect Dis. 2023 Jan;29(1):e221025. doi: 10.3201/eid2901.221025 (PMC9796222; doi:10.3201/eid2901.221025)
Supplement: Appendix — Additional information for deployment, management, and performance of field laboratories for Ebola virus disease outbreak during chronic insecurity, eastern Democratic Republic of the Congo, 2018–2020. [file 22-1025-Techapp-s1.pdf]

Article DOI: https://doi.org/10.3201/eid2901.221025

Efficiency of Field Laboratories for Ebola Virus Disease Outbreak during Chronic Insecurity, Eastern Democratic Republic of the Congo, 2018–2020

Appendix

1: Decision-making for laboratories' deployment

Key factors influencing whether and where to deploy field laboratory

1) Increase of confirmed-cases in a defined area not previously reporting cases

2) Lack of pre-existing laboratory capacity in the affected area

3) Possibility of EVD outbreak to reach big cities, from where it can easily spread (presence of roads, airports, harbors…)

4) Population density in the affected area (as this can accelerate the EVD spread)

5) Possible population movements (especially if roads were in good conditions)

6) Possibility of crossing internal and international borders

7) Risk of spread to an unsecured zone (land under rebels' control)

8) Site accessibility of samples shipment and laboratory's supplies refill

Additional points addressed before proceeding to deployment

1) Proximity to EVD treatment units

2) Easy access for surveillance and safe and dignified burials teams

3) Presence of public health facilities in the area to host the laboratory

4) Availability of rooms constructed in durable materials (well ventilated with water/electrical supply, ready to be reconfigured into the desired standards)

5) Presence of an incinerator for biohazardous waste

6) Security measures including an integral fence and guards/units

2: Main challenges

Challenging situations can be summarized as follows:

A. Related to access and security issues

1) Bad road conditions in certain areas
2) Harsh geographical conditions to access certain areas
3) Cost of transport
4) Safety and security threats and attacks on personnel (from rebels, militia and population), equipment (loss or damage) and work (have to stop working several hours even)

B. Work force issues

1) Need of building capacity for local staff in lab techniques such as working in the glovebox, unidirectional flow work, PPE use, decontamination process
2) Biosafety and biosecurity practices (almost new for most of staff)

C. Logistics issues

1) Limited storage capacity due to important number of samples processed per day (13 laboratories) and also samples stuck in laboratories during insecurity periods
2) Difficulty to organize more shipments from the field due to high cost of chartered flights
3) Related to equipment maintenance, damage and loss
4) Related to energy requirements

D. Related to data management & communication of results

E. **Financial resources issues:** limited financial resources due to steadily increase in human resources, laboratory's needs (reagents and supplies shortage) and logistics to support

3: Waste management

1) Non-biohazardous waste was collected in plastic bags which were wrapped, then sealed with adhesive tape until they were transported to the incinerator.
2) Laboratory waste was treated as biohazardous and separated at each step of samples processing.
3) Items soiled by biological materials (tubes, tissues, pipet-tips etc) were processed in the glovebox and disinfected with 10% bleach or others (Rely+On Virkon®, CDiffend®).
4) After samples' manipulation, soiled materials were wrapped in small biohazard bags, sealed with adhesive tape, sprayed with disinfectant to minimize the microbial load, taken out of the glovebox and dropped into larger biohazard bags which, were also sealed with adhesive tape.
5) Trained hygienists used plastic garbage to transport all parcels and load them into the incinerator.
6) Used Xpert® Ebola cartridges were stored across sites and then shipped to the central repository in Beni for temporary storage.
7) Once the Addfield® incinerator (could reach > 1000°C) was purchased and installed in July 2019, all used Xpert® Ebola cartridges were regularly shipped to Goma for appropriated disposal.

4: Power management

1) Electricity was continuously supplied by 10 to 50 Kilo volt Ampere (KVA) generators.

2) Equipment was connected to Uninterruptable Power Supply (UPS) and stabilizers units to prevent any electricity variation.
3) Backup generators were alternately run to ensure uninterrupted power supply to the laboratory.
4) Management of the generators required an extensive logistics, continuous fuel, oil and spare-parts provision for almost two years.

5: Genomic sequencing

1) At the beginning of the outbreak, the first samples of confirmed cases were regularly shipped to INRB-Kinshasa for full genomic sequencing.
2) From September 2019, we implemented a field genomic sequencing laboratory in Katwa (Butembo city) to support the surveillance activities in the monitoring of an epidemic which tended to get out of control (spread in the time and the space) with new confirmed cases emerging from unidentified sources.
3) The near real-time data generated throughout the outbreak helped in the detection and management of unknown transmission chains.
4) One aliquot of samples from new confirmed cases was transferred from different sites to the sequencing laboratory in Katwa.
5) The remaining aliquots were rapidly shipped with Ebola negative samples to the central cold chain in Beni.
6) The maximum time taken to transport positive aliquots from their laboratory of confirmation to the sequencing laboratory was 48-72 hours, following the availability of transport means (helicopter/plane flights and vehicles).
7) The field sequencing laboratory analyzed 219 samples in total, 60 of them did not have any epidemiological links although 39 of them (65%) gave full genome coverage.

6. Field Laboratory Deployment Checklist

Product ID	Category	Description	Unit	Note
Laboratory Equipment				
	Laboratory Equipement	GeneXpert Machine	each	
	Laboratory Equipement	UPC	each	
	Laboratory Equipement	Piccolo Analyser	each	
	Laboratory Equipement	ISTAT Analyser	each	
	Laboratory Equipement	Sysmex Analyser	each	
	Laboratory Equipement	Fish Box/ Glove box/Hepa Filter Cleateach Box	each	
Office Equipment				
	Office Equipement	Printer	each	
	Office Equipement	Lap top	each	
Cold chain Equipment				
	Cold chain Equipement	Freezer - 20 or -80°C	each	If possible
	Cold chain Equipement	Refrigerator +2 à 8° C	each	
Laboratory Supplies				
	Laboratory Supplies	DuPont Tyvek 400 Coveralls with Respirator Hood, cs	Case	
	Laboratory Supplies	Face Shield, Comfort Band, 100/cs	Case	
	Laboratory Supplies	GeneXpert CE-IVD 50	Case	
	Laboratory Supplies	Gown Surgery Sterile, XL 100/Case	Case	
	Laboratory Supplies	Kimberly-Clark Professional Wypall L10 Utility Wipers 18/case	Case	
	Laboratory Supplies	Lab Coats - Medium, cs	Case	
	Laboratory Supplies	Lab Coats - X-large, cs	Case	
	Laboratory Supplies	Lab Coats - Large, cs	Case	
	Laboratory Supplies	Nitrile Glove, Powder Free, Latex Free, XL, 100/pk	Case	
	Laboratory Supplies	Nitrile Glove, Powder Free, Latex Free, LG, 100/pk	Case	
	Laboratory Supplies	Nitrile Glove, Powder Free, Latex Free, Med, 100/pk	Case	
	Laboratory Supplies	Nitrile Glove, Powder Free, Latex Free, Sm, 100/pk	Case	
	Laboratory Supplies	Piccolo Amlyte 13 Panel (10 disk/Box)	Box	

	Laboratory Supplies	Piccolo Comprehensive Metabolic Panel Disk	Box	
	Laboratory Supplies	Premium 81- Place Cardboard Box, Deep Lid	case	
	Laboratory Supplies	Removable Cover Racked LTS Tips 1000UL in Racks	5 box per case	
	Laboratory Supplies	Removable Cover Racked LTS Tips 200UL in Racks	5 box per case	
	Laboratory Supplies	Removable Cover Racked LTS Tips 10UL in Racks	5 box per case	
	Laboratory Supplies	Shoe Cover IsoClean LG 100/cs	Case	
	General Supplies	Specimen collection Cups (Semen Specimen)	Case	
	Laboratory Supplies	Sysmex Poch Pack D 2x2L	Pack	
	Laboratory Supplies	Sysmex Poch Pack L 2x250 ml	Pack	
	Laboratory Supplies	Ultrapure Water 500 ml	each	
	Laboratory Supplies	K2EDTA Blood Collection Tubes, 2mL 100 /rack	rack	
	Laboratory Supplies	Gown Surgery Sterile, Large 100/case	Case	
	Laboratory Supplies	Pen Permanent Marker Blaack sharpie 10/box	Box	
	Laboratory Supplies	Thermal Results Printer Paper Piccolo Xpress, 6 rolls	Box	
	Laboratory Supplies	Sysmex Thermal Paper for POCHI-100i Analyzer, 3 rolls/pk	Pack	
	Laboratory Supplies	iSTAT CK-MB Cartridges, 25/bx	Box	
	Laboratory Supplies	iSTAT CHEM8+ Cartridges, 25/bx	Box	
	Laboratory Supplies	Sysmex Poch Pack 65XL 9L	Pack	
	Laboratory Supplies	CDIFFEND Tablets - 2XL	Tablet	
	Laboratory Supplies	Rely+On Virkon™	Pack	
	Laboratory Supplies	2mL Sarstedt Tubes,	each	
	Laboratory Supplies	3M 6969 Duct Tape Sliver,	each	
	Laboratory Supplies	3M Aura Particulate Respirator, N95 or surgical face mask	each	
	Laboratory Supplies	BD Vacutainer Plastic Blood Collection Tube w K2EDTA, 4mL	each	
	Laboratory Supplies	BD Vacutainer Plastic Blood Collection Tube W Lithium Heparin, 4mL	each	
	Laboratory Supplies	Biohazard Bag 38x48" 55GL, 100/case	each	
	Laboratory Supplies	Biohazard Bag, 20-30G, 200/case	each	
	Laboratory Supplies	Burn Boxes, 6/pk	Pack	
	Laboratory Supplies	iSTAT CG8+ Cartridge,	Box	
	Laboratory Supplies	Triple packaging	Box	
	Laboratory Supplies	Virocult 125 /case	Case	
	Laboratory Supplies	Bleach 1,5L	each	

	Laboratory Supplies	Blood collection set +older G21; or G23 240/case	each	
	Laboratory Supplies	Urine/semen collection Cups	each	
	Laboratory Supplies	Ethanol 96 % 5l	Can	
